# Experiences of using a digital tool, the D-foot, in the screening of risk factors for diabetic foot ulcers

**DOI:** 10.1186/s13047-022-00594-9

**Published:** 2022-12-13

**Authors:** Roland Zügner, Gustav Jarl, Leif Sundberg, Ulla Hellstrand Tang

**Affiliations:** 1 Department of Orthopedics, Institute of Clinical Sciences, Sahlgrenska Academy, University of Gothenburg, Sahlgrenska University, SE-413 45 Göteborg, Sweden; 2 Forskningsenhet Ortopedi, Göteborgsvägen 31, SE-431 80 Mölndal, Sweden; 3 Department of Prosthetics and Orthotics, Faculty of Medicine and Health, Örebro University, Örebro, Sweden; 4 University Health Care Research Centre, Faculty of Medicine and Health, Örebro University, Örebro, Sweden; 5 Gothenburg Diabetes Association, Gothenburg, Sweden; 6 Department of Prosthetics and Orthotics, Sahlgrenska University Hospital, Gothenburg, Sweden

**Keywords:** Diabetic foot, Foot ulcer, eHealth, Orthotics, Foot orthotics, Insole, Shoes, Implementation, diabetes, foot deformities

## Abstract

**Background:**

Individuals living with diabetes run an increased risk of developing diabetic foot ulcers (DFUs), leading to high costs to society and reduced quality of life for the individual. Regular screening is important to avoid complications.

**Aim:**

To evaluate patients’ and clinicians’ experiences of using a digital tool, the D-Foot, in the screening of risk factors for developing DFUs. The secondary aims were to investigate whether patients had had their feet examined by a nurse or doctor during the past year, had been referred to podiatry and whether patients had received information about self-care.

**Methods:**

A prospective study was carried out, comprising 90 patients with diabetes visiting a Department of Prosthetics and Orthotics (DPO). Two Certified Prosthetists and Orthotists (CPOs) were included, and they assessed foot status and the risk of developing DFUs with the D-Foot software, prior to prescribing footwear. The quality of services at the DPO was assessed by the patients using the Orthotics and Prosthetics Users’ Survey (OPUS). The CPOs answered the System Usability Scale (SUS) before and after the study to assess the usability of the D-Foot.

**Results:**

No patient had risk grade 1. One (1%) patient had risk grade 2, 78 (87%) patients had risk grade 3 and 11 (12%) patients had risk grade 4. Patients reported high levels of satisfaction on eight of ten OPUS items and the two items with lower scores were not related to the use of the D-Foot. The two CPOs reported levels above the mean regarding usability both before (77.5 and 90) and after (70 and 97.5) using the D-Foot.

**Conclusions:**

Patients expressed a high level of satisfaction with the services when their feet were examined with the D-Foot prior to the provision of footwear. The CPOs found that the D-Foot system was usable. Several comments were made by patients and CPOs and will support the future development and testing of the D-Foot. There is a need to increase referrals for preventive podiatry and improve information on self-care for patients at risk of DFUs.

**Trial registration:**

ClinicalTrials.gov ID: NCT04054804.

**Supplementary Information:**

The online version contains supplementary material available at 10.1186/s13047-022-00594-9.

## Background

Individuals living with diabetes run an increased risk of developing complications such as diabetic foot ulcers (DFUs) and this complication might lead to an amputation [1]. In 2021, the total number of patients at risk of developing DFUs was estimated at 270 million globally [2] and 250,000 in Sweden [3–5], given that 50% of the patients have peripheral neuropathy [6, 7]. DFUs are associated with poor quality of life [8] and high costs; the annual cost/DFU has been estimated at 19,000 US$ (around 110,000 SEK) [9–11]. The prevention of DFUs is essential to preserve the quality of life of patients and reduce healthcare costs to society [12–14]. The prevention of DFUs should include: 1) regular screening for risk factors for developing DFUs, 2) intervention with prescribed footwear (shoes and/or insoles), 3) podiatry and 4) patient self-care education [15–18]. As stated by the World Health Organisation, therapeutic footwear is necessary for persons with diabetes with peripheral neuropathy and risk factors for developing DFU. Prescribed footwear is one of 50 assistive devices that should be available for those in need, as recommended by the World Health Organisation in the Priority Assistive Products List [19].

The opportunities for introducing new clinical examination methods, such as a digital tool, need to consider how the healthcare system is organised and the laws, regulations and guidelines when examining and prescribing assistive devices to patients with diabetes at risk of developing DFUs [20]. Some of the laws that form care in Sweden are: a) the patient law that states that care should be patient centred and that healthcare professionals should have a dialogue with the patient regarding examination, treatment and a care plan [21] and b) the law relating to the need to consider the integrity of the patient when data are registered in the medical record systems [22, 23]. These laws aim to promote the patient’s integrity, self-determination and participation and must be followed by all healthcare professionals [22, 23]. When implementing new eHealth systems, the European regulations regarding the development and maintenance of a medical product need to be followed [24].

Starting in 2010, the D-Foot was developed by healthcare professionals, patients and researchers in Sweden with the aim of: 1) facilitating foot screening, 2) generating an objective risk stratification (1 = no risk, 2 = low risk, 3 = medium risk and 4 = ongoing DFU) and 3) individualising care and advice regarding self-care with the aim of promoting good foot health and preventing DFUs. A detailed description of the D-Foot is presented in the method section. The D-Foot was designed to be used primarily by clinicians working at DPOs [25] and secondarily by other healthcare professionals to improve the care of patients at risk of developing DFUs [26, 27].

It has been suggested that digital tools, such as the D-Foot, improve DFU management in care [28–31] and stored data can be useful when auditing the care of DFU patients to support clinical improvements, as presented by Leese et al. [30]. Continuous improvements based on the users’ experiences to develop and implement improvements are necessary [32, 33].

The primary aim of the study was to evaluate the users’ experiences of using the D-Foot in the screening of risk factors for developing DFUs. The secondary aims were to investigate whether patients had had their feet examined by a nurse or doctor during the past year, had received podiatry and whether the patient had been given information about self-care.

## Methods

### Study design and procedure

**Fig. 1:**
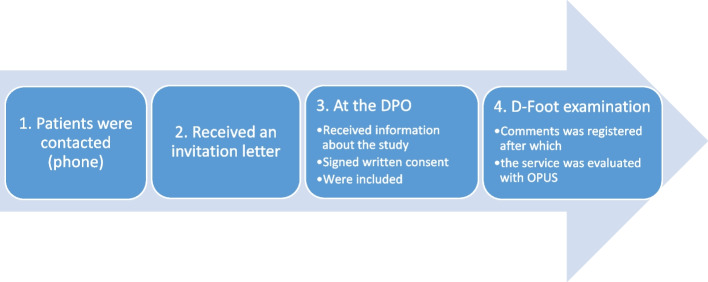
A visualisation of the different steps in the study. *Note*: OPUS; Orthotics and Prosthetics Users’ Survey (OPUS) [34]

This prospective study was carried out in 2019 in a cohort of patients with diabetes referred to the DPO at Sahlgrenska University Hospital in Gothenburg, Sweden. The purpose of the visit to the DPO was to provide the patient with pressure-relieving footwear, insoles together with shoes, to counteract the occurrence of DFU. The referred patients were contacted by phone and patients interested in participating in the study were informed by the principal investigator and received an invitation letter to the DPO, Fig. 1. The letter included information on how to answer the patient questionnaire in the D-Foot, Additional file 1. The second way for the patients to answer this questionnaire was at the DPO, where the survey was visualised on a tablet (Samsung Galaxy Tab. A 10.1). The patients’ answers were registered before the clinical examination began. If needed, the investigator helped the patients to register their answers on the tablet. The clinical examinations were assessed by one of two CPOs, at the DPO, following the routine in the D-Foot. The daily clinical work process in the prescription is as follows: a) the CPO evaluated the patients’ need for prescribed footwear, b) suggested and discussed a care plan with the patient, c) gave self-care advice and d) provided the patient with footwear as described in clinical guidelines [35]. The information, advice and provision of footwear were based on the patient’s risk of developing DFUs according to the D-Foot examination. Finally, the D-Foot assessment summary was printed out as a PDF report and given to the patient. The footwear was delivered either on the first visit or on a second visit to the DPO, two to 8 weeks later, Additional file 9.

The CPOs documented the assessments, tests, care plan decisions and the information they gave to patients in the local EMR system, Pilot, used at the DPO situated in Region Västra Götaland, Sweden.

The patients’ experiences of the visit to the DPO and the D-Foot examination were evaluated using the Orthotics and Prosthetics Users’ Survey (OPUS) [34]. The survey was filled in after the patients had been provided with their prescribed footwear. The users’ experiences of using the D-Foot were explored by registering the users’ (patients and CPOs) comments as they used the D-Foot at the DPO, Fig. 1. The method used was the think-aloud method, a method frequently used in usability testing where the spontaneous comments from the users are registered, in this case the patients and CPOs [36]. The registration was made by the principal investigator.

The CPOs’ expectations and experiences of using the D-Foot were evaluated using the System Usability Scale (SUS), before and after the study period.

### Study sample – CPOs

Two experienced CPOs at the DPO at Sahlgrenska University Hospital agreed to participate in the study. They were skilled in prescribing footwear to patients at risk of developing DFUs with assistive devices and had worked as CPOs for nine and 20 years respectively.

Before the start of the study, each CPO completed a D-Foot introductory course which lasted for 2 h on two separate occasions. Moreover, the CPOs participated in a workshop with the purpose of becoming familiar with the D-Foot examination routine.

### The D-foot software

The D-Foot software has seven functions: 1) a booking system to schedule patients’ appointments, 2) a questionnaire to be answered by patients, 3) examinations to be completed by a healthcare professional, Additional files 2, 4) a system for generating a risk classification for each patient, Additional files 3, 5) a summary as a PDF report, 6) a database containing all registered information and 7) an administration part licensing the CPOs to obtain access to the program [25]. A brief description of some different components included in the D-Foot is also included in Additional file 4 and a summary in Additional file 10. A detailed description has been presented in a previous study by Hellstrand Tang et al. [25]. Based on the results from the previous study, the users (eight CPOs) [25] suggested refinements as compared with the first version. The following improvements were therefore made: 1) adding a test of signs of peripheral angiopathy with the question “*Has a healthcare professional confirmed that you have peripheral angiopathy?”*, 2) excluding the navicular drop test [37], 3) excluding the test entitled “Can you extend and flex you toes”, 4) excluding the question of whether the foot had areas of excessive pressure with calluses, 5) excluding the assessment “Gait deviation, affected from hip/knee joint” and 6) splitting the assessment of Charcot foot (acute Charcot foot and manifest deformed Charcot foot) [25].

Furthermore, in the current version, the order of the assessment was reorganised to fit more effectively into a more clinical flow with the patient being examined first in a sitting position, followed by standing assessments.

Based on the patients’ answers in the survey and the assessment made by the CPO, a risk classification (1–4) was generated according to the national risk classification system [26, 38], Additional file 3. In this risk classification, the symptoms that are related to each of the risk categories are presented: peripheral neuropathy/angiopathy, foot deformities, skin pathologies, previous DFU/amputation, active DFU/Charcot deformity and/or severe pain syndrome. The risk classification, together with the recommended interventions, such as podiatry, footwear, and regular foot examinations, was automatically displayed on the screen of the tablet and was also included in the PDF report that was given to the patient. Finally, the CPO copied the results from the D-Foot and pasted them into the patient’s EMR and, in addition, all the data were stored in a separate D-Foot database. The reliability and validity of the D-Foot software have previously been presented by Hellstrand et al. [25].

### Equipment

The CPOs recorded their findings on a laptop. Patients answered the patient questionnaire using a smart phone, tablet, or personal computer. A goniometer was used to measure passive maximum dorsal flexion at the metatarsal phalangeal joint and passive dorsiflexion at the ankle joint, as previously described by Hellstrand Tang et al. [25]. A foot calliper was used to measure foot length and foot width, while toe height was measured with a ruler [25], Additional file 4.

### Questionnaire – patients

The patients answered the Swedish version of the Client Satisfaction with Services module of the OPUS after they had received their footwear. The OPUS module assesses patients’ experiences of the service quality and has been validated with Swedish patients at DPOs [34]. The 10 items are answered on a three-level Likert scale (disagree = 1, agree = 2 and strongly agree = 3) and were analysed by calculating the mean and standard deviation for each question. The OPUS questionnaire is recommended by the Swedish Orthotic and Prosthetic industry advisory council to evaluate patient satisfaction with the services at DPOs located in Sweden [39].

### Questionnaire – CPOs

In connection with the start of the study, a Swedish version of the System Usability Scale (SUS) was answered by the CPOs to capture their expectations of using the D-Foot in clinical work, Additional file 5. The SUS consists of 10 questions answered on a five-level Likert scale (from strongly disagree = 1, to agree completely = 5) and is a reliable tool for measuring usability [40]. At the end of the study, the CPOs answered the SUS a second time to assess their experiences of using the D-Foot, Additional file 6. These answers were transformed to a 0–100 scale, where a higher score indicates a higher level of usability.

In addition, at the end of study, the CPOs answered a study-specific questionnaire on how they had experienced the digital foot examination, what information they had given to the patients and how long it took for the CPOs to execute different sequences of the examination, Additional file 7. The questions on time estimations were related to how long it took to: 1) finalise the patient appointment and foot assessment when the D-Foot routine was used (< 30 min., 31–45 min., 46–60 min., > 60 min), 2) make the medical recording based on the D-Foot examination (< 6 min., 6–10 min., 11–15 min., 16–20 min.) and 3) order their prescribed footwear (< 6 min., 6–10 min., 11–15 min., 16–20 min.), Additional file 8.

### Statistics

Descriptive statistics were used to present patient demographics. The mean and standard deviation were calculated for continuous variables. Differences between the included patients and the non-included patients were compared with an independent t-test for age and Pearson’s chi-square test for gender. IBM SPSS Statistics for PC, Version 25, was used for all statistical calculations.

## Results

### Study sample – patients

Patients on the waiting list, *n* = 107, at the DPO, Sahlgrenska University Hospital, with a referral to prevent foot complications in diabetes, were invited to participate in the study. The inclusion criteria were being diagnosed with diabetes, age ≥ 18 years and understanding the Swedish language.

The studied group finally consisted of 90 patients, 53 men and 37 women, with a mean age of 68 ± 13 years. The patients who were not included (*n* = 17), nine men and eight women, had a mean age of 64 ± 15 years and did not differ significantly from the included participants according to gender (*p* = 0.65) and age (*p* = 0.27). Of the entire group, 57 patients had retired and 17 were working, Table 1.

**Table 1:** Descriptive data of included patients

Categorical and continuous variables	n (%)	Mean (SD)
Women	37 (41)	
Type 1 diabetes	33 (37)	
Type 2 diabetes	55 (61)	
Diabetes, other types	2 (2)	
Duration, yrs		21 (16)
Age, yrs		68 (13)
HbA1c, mmol/mol, self-reported		62 (19)
Height, m		1.72 (0.9)
Weight, kg		83 (20)
BMI, kg/m^2^		28 (6)
Medication for high blood pressure	61 (68)	
Medication for heart disease	45 (50)	
Smoker	6 (7)	
Use snuff	9 (10)	
**Occupation**		
Working	17 (19)	
Students	0 (0)	
Retired	57 (63)	
Other	6 (7)	
Missing value	10 (11)	

The examination showed that no patient had risk level 1, one (1%) patient had risk level 2, 78 (87%) patients had risk level 3 and 11 (12%) patients had risk level 4. At the examination, the patients were asked about the interventions to prevent DFU they had received. Seventy-eight (87%) patients had had their feet examined by a nurse or doctor during the past year, 59 (66%) answered that they had received podiatry and 59 patients (66%) had been given information about the self-care of their feet.

### Orthotics and prosthetics users’ survey-OPUS

Eighty-two (91%) of 90 patients answered OPUS. The mean value of the questions ranged from 2.3 to 2.9, where 2 = agree and 3 = strongly agree, Table 2. Satisfaction with services was highest for the questions related to how the CPOs showed courtesy and respect, how the patients were informed about the choice of footwear and the opportunity to express their own concerns. Satisfaction was lowest for the waiting time for the appointment and the co-ordination of services.

**Table 2:** Results from the orthotic and prosthetics users’ survey (*n* = 82)

Item	Mean (SD)	Not applicable, n (%)	Missing, n (%)
I received an appointment with a prosthetist/orthotist within a reasonable amount of time.	2.3 (0.8)	0	0
I was shown the proper level of courtesy and respect by the staff.	2.9 (0.3)	0	0
I waited a reasonable amount of time to be seen.	2.8 (0.4)	0	1 (1)
Clinic staff fully informed me about equipment choices.	2.9 (0.3)	7 (9)	0
The prosthetist/orthotist gave me the opportunity to express my concerns regarding my equipment.	2.9 (0.4)	1 (1)	0
The prosthetist/orthotist was responsive to my concerns and questions.	2.8 (0.4)	7 (9)	0
I am satisfied with the training I received in the use and maintenance of my prosthesis/orthosis.	2.7 (0.5)	19 (23)	0
The prosthetist/orthotist discussed problems I might encounter with my equipment.	2.7 (0.5)	14 (17)	0
The staff co-ordinated their services with my therapists and doctors.	2.3 (0.6)	39 (48)	0
I was a partner in decision-making with clinic staff regarding my care and equipment.	2.8 (0.4)	3 (4)	1 (1)

*Note*: Rating scale: disagree =1, agree = 2 and strongly agree = 3

### System usability scale

One CPO had an SUS score of 77.5 before using the D-Foot and 70.0 after the study was completed. The second CPO had an SUS score of 90.0 before using the D-Foot and 97.5 after the study was completed, Table 3.

**Table 3:** Results from the System Usability Scale (SUS) answered by two CPOs

Questions, before the study start	CPO 1 before (after)	CPO 2 before (after)
1. I think that I would like to make a digital assessment.	4 (4)	5 (5)
2. I think I will find that a digital foot assessment is unnecessarily complex.	2 (1)	1 (1)
3. I think I will find that a digital foot assessment is easy to perform.	4 (4)	5 (5)
4. I think that I will need the support of a technical person to be able to make a digital assessment.	4 (3)	5 (2)
5. I think I will find that the various functions for performing digital assessments are well integrated.	4 (3)	5 (5)
6. I think I will find that there is too much inconsistency in the digital assessment.	2 (2)	1 (1)
7. I think that most people will quickly learn how to make a digital assessment.	4 (4)	4 (5)
8. I think I will find making digital assessments very complicated.	2 (2)	1 (1)
9. I think I will feel very confident about making a digital assessment.	4 (4)	5 (5)
10. I think I will need to learn a lot of things before I can make a digital assessment.	2 (3)	4 (1)
Total SUS score per observer	77.5 (70.0)	90.0 (97.5)

*Note*: The questions that are presented in this table were answered by the CPOs prior to study start. The questions in the SUS that were answered after the study was completed are described in Hellstrand et al. 2017 [31]

Low scores to the questions with an even number should be interpreted as positive, as the user somewhat disagrees (strongly disagree = 1 to strongly agree = 5)

The original data are presented in the table. Thereafter, the total SUS score was calculated as follows: each question was answered with a score (strongly disagree = 1 to strongly agree = 5). From the answers with an odd number, “one” was subtracted. Thereafter, from the answers with an even number, the number was subtracted from “five”. The value obtained per person and question ranged from 0 to 4, with four as the highest response. The sum was based on the converted numbers multiplied by 2.5 and a summary score for all 10 questions, ranging from 0 to 100, was obtained [40].

The 10 questions in the SUS have been translated into Swedish, from the original English text. The Swedish response format was: 1) Håller absolut inte med, 2) Håller inte med, 3) Håller varken med eller inte med, 4) Håller med and 5) Håller fullkomligt med

### Comments and feedback from patients and CPOs

In total, 147 comments were registered, (*n* = 50 from patients and *n* = 97 from CPOs), Table 4. Overall, 55 comments were related to improvements (*n* = 26 from patients and *n* = 29 from CPOs). Patients suggested refinements to the patient questionnaire regarding the perceptions of neuropathy; pain, previous ulcers, sweat and HbA1c. Patients found questions such as “Have you had a foot ulcer? and “Are your feet less sweaty now compared with recent years?” difficult to answer. Patients suggested specifying the question of “Have you previously had hard-to-heal ulcers?” and removing the question about sweaty feet, because some patients said that they had never had sweaty feet. Further, they suggested expanding the question on the sensation of tingling or numbness to include sensations of cramp, walking on pillows and feelings such as “it feels like a band around my foot”. The question about the presence of pain should be more specific, such as “How much pain have you experienced in your feet during the past week”.

**Table 4:** Summarised comments (*n* = 51) from the patients and the CPOs registered as they used the D-Foot

Category of comments	Frequency comments	
	Patients (*n* = 50)	CPO (*n* = 97)
D-Foot improvements	26	29
Medical record system not functioning	1	14
Patient was not booked in D-Foot alongside with the booking in the electronic medical recording system at the DPO	6	11
New way of working	1	5
New way to access care	7	1
No access to patient survey from home	2	4
Patients identity and integrity	0	7
Problem with the tablet	0	3
Difficult to change booking in the D-Foot	0	2
Services at DPO	4	1
Wi-Fi not functioning	0	2
Technical equipment	0	4
Technical error with CPOs’ laptop	0	2
Technical problems	0	2
Double-booked in the D-Foot	0	2
Patient not familiar with the Swedish language	2	1
Bad ergonomics to work with a small laptop	0	1
A complicated booking system in the D-Foot	0	4
Problems with the D-Foot web program	1	0
Problems with the study setting	0	2

*Note*: DPO, departments of prosthetics and orthotics

*CPO* Certified Prosthetist and Orthotist

Other suggested improvements, coming from both patients and CPOs, were to design the PDF report in an easy-to-understand format with larger text, linking the risk grade to each of the specific risk factors and, in addition, creating customised information and advice about self-care of the feet for patients. Requests were also made to be able to post the report in digital format in addition to the printable version. The individual risk factors should be linked to the recommended treatment (e.g. recommendation to use a high toe box in the presence of hammer toes). Moreover, digital referrals to other healthcare professionals, based on the results, should be included in upcoming versions.

The CPOs requested integration with co-working systems, such as the population register, the EMR used by other healthcare professionals and the EMR used at the DPO. Loss of wi-fi, breakdown of the EMR at the DPO and problems registering findings in the D-Foot system were registered. Eleven patients were not booked in the D-Foot system and these patients were therefore unable to answer the patients’ questions at home, prior to the visit.

Of the 90 patients, 18 (20%) answered the patient survey prior to the visit to the DPO and 72 (80%) answered the survey on a tablet at the visit. More than 50% (*n* = 44) were helped at the DPO to answer the survey.

### CPOs’ experiences of the digital foot examination and information to the patient

The CPOs felt that they had provided “complete” information regarding a) how patients should perform self-care for their feet, b) where patients could obtain additional information, c) potential risks when using the assistive device and d) warning signs relating to health and the assistive device. Regarding the questions: a) whether the examination took place in privacy, b) information on the D-Foot results and c) whether the patient had received oral and written information about the assistive device, they were judged to have been performed “completely”. Two questions were judged to be less well fulfilled by one of the CPOs: a) the discussion of the health condition and b) whether the results of the D-Foot survey were explained in a way the patient understood, Table 5.

**Table 5:** Answers from the certified prosthetists and orthotists

Questions	1. *Not at all*	2.	3.	4. Y*es, completely*	5. *Not applicable*
*Did the patient receive enough information about how to make self-care of the feet?*				2	
*Was the illness/health condition discussed?*			1	1	
*Did you leave information about where to go if the patient needed help or had additional questions after the visit?*				2	
*Did the patient receive enough information about possible risks with using the assistive device?*				2	
*Did the patient receive enough information about warning signs to be aware of regarding his/her illness/health condition or your assistive device?*				2	
*Was there enough privacy when you and the patient discussed his/her condition or treatment?*				2	
*Did you explain the results of the D-Foot survey in a way that the patient understood?*		1		1	
*Did you give the patient oral user information about the assistive devices?*				2	
*Did you give the patient written information about the assistive devices?*				2	

The estimated time required for different sequences when meeting the patients was assessed equally by the two CPOs. The time required for the patient appointment was 40–60 minutes. Writing the medical record took six to 10 minutes and ordering shoes and materials took six to 10 minutes. The patient’s visit was finalised within a total of 52–80 minutes.

## Discussion

This is the first implementation study of a digital screening tool, the D-Foot, and we found that patients were generally satisfied with the services when being routinely examined using the D-Foot. The CPOs found the D-Foot software usable. It is worth noting that one third of the patients reported that they had not received podiatry or received information about foot self-care, which is concerning.

The patients generally expressed a high level of satisfaction with the services at the DPO when using the D-Foot for the foot examination. It is worth noting that not all the OPUS items relate to the foot examination and the two items with the lowest scores (waiting times and co-ordination of services) relate to the items that were not related to the foot examination and the use of the D-Foot.

The usability, according to the SUS, was generally high both before and after using the D-Foot, as scores above 68 can be regarded as “above average” [40, 41]. For one CPO, the SUS score improved by 7.5 points after having used the D-Foot, indicating that this CPO found the D-Foot’s usability better than he had expected prior to using it. For the other CPO, the SUS score deteriorated by 7.5 points after having used the D-Foot. This is most likely explained by technical problems with the wi-fi system. However, on individual items, most ratings were similar before and after using the D-Foot. Only two ratings by one of the CPOs changed by more than one step on the rating scale. One expectation changed in both the positive and negative direction after the CPOs had used the D-Foot. One CPO responded more positively (from 5 = strongly agree to 2 = do not agree) regarding “I think that I will need the support of a technical person to be able make a digital assessment”, indicating that, by using the D-Foot, the need for technical support decreased. Several other questions were scored positively both prior to and after use, such as the D-Foot was easy to use, it was quick to learn, and the user felt confident about using the D-Foot.

A variety of comments were collected from the CPOs and these comments from users are useful in the continuous improvement of digital tools such as the D-Foot software [32]. In future versions, the authors suggest that improvements should be made. For example, to specify the question regarding pain as “*How much pain as a mean value did you perceive last week in your right foot*”, followed by a Likert scale. Furthermore, a more easy-to-understand report, with custom-made information regarding footwear and self-care, would make it easier for persons with diabetes to perform self-care based on their individual risk factors. The report should be available in both a printed and a digital version, with the option of being sent to the patient.

Continuous improvements, based on the needs of different healthcare users, are a necessity, as described by Koltveit et al., when implementing digital tools in the care of DFUs [33]. The authors suggest that digital tools, such as the D-Foot, are useful when implementing seamless and person-centred care for persons living with diabetes with a risk of foot complications, as recommended by the Swedish Association of Local Authorities and Regions [42].

Several improvements were suggested by the patients and CPOs, such as clarifying and differentiating the patients’ questions and facilitating the booking system by connecting the D-Foot to the population register. Patient security and integrity will definitely be improved by integration with the population register. This would ensure that the right patient with the right identity is booked in the D-Foot system. Integration with the EMR used by other healthcare professionals would lead to data being available where the patient is treated: in primary care, in specialist care or at the DPO. Moreover, with integration between systems, digital referrals and responses to referrals would improve communication, thereby improving the quality of care. To summarise, the D-Foot is useful for CPOs and would improve the quality of care without requiring more time for each visit.

Unstable wi-fi is a concern, as presented in this study. In addition to developing functioning software, the D-Foot, the DPOs need to assure that wi-fi is in place.

Secondly, the use of the stored D-Foot is useful for audit and follow-up at local, regional and national level and fills a gap identified in national guidelines [20].

A default value of “no risk factor” was suggested by one CPO as a way of facilitating the CPOs’ assessment. If a risk factor is present, the CPO registers this specific risk factor.

Patient satisfaction with the service at the DPO revealed relatively high scores according to the results from OPUS. Two questions were answered as “not applicable” and the reason might be that patients do not regard footwear (insoles and shoes) as assistive devices. This semantic question needs to be clarified and specified as shoes and insoles, to avoid misunderstanding.

One limitation of the study was that no control group of patients who received traditional examination of the feet was used. As a result, it is not possible to determine the extent to which the patients’ high level of satisfaction with the services was due to the use of the D-Foot or to the general appreciation of the services at the DPO. Only two CPOs were included, limiting the interpretations of the CPOs’ answers regarding the SUS. A larger sample is recommended in future studies. The two CPOs only answered a study-specific questionnaire at the end of the study. This evaluation, Appendix 4, should preferably be carried out after each D-Foot assessment made for each of the participants. This could not be carried out after each patient, due to the daily CPO-scheduled work. Finally, the study was conducted at a single DPO, which limits the generalisability of the results, and future studies should include CPOs at different locations.

## Conclusion

Patients with diabetes expressed a high level of satisfaction with the services when their feet were examined following the routine in the D-Foot software prior to the provision of footwear. The CPOs felt that the D-Foot system was usable. Several comments were made by patients and CPOs and will support the future development of and improvements to the D-Foot software. The current study revealed that not all patients in need had access to preventive interventions such as podiatry and information about self-care.

## Supplementary Information

**Additional file 1. ** Patient survey included and programmed in the D-Foot.

**Additional file 2. **Examples of different steps in the D-Foot examination.

**Additional file 3. **Prevention and multidisciplinary service (MDS) of foot complications in diabetes.

**Additional file 4.**

**Additional file 5. **System Usability Scale answered prior to the study.

**Additional file 6. **System Usability Scale answered after the study.

**Additional file 7. **Questions related to the information the certified prosthetist and orthotist gave to the patients and questions related to integrity and time to finalise the visit.

**Additional file 8.**

**Additional file 9.**

**Additional file 10.**

## Data Availability

The datasets used and/or analysed during the current study are available from the corresponding author in response to a reasonable request, if the appropriate permits are obtained from the correct authorities.

## Abbreviations

DFUs: Diabetic foot ulcers
DPO: Department of Prosthetics and Orthotics
CPOs: Certified Prosthetists and Orthotists
OPUS: Orthotics and Prosthetics Users’ Survey
SUS: System Usability Scale
EMR: Electric medical recording
US$: US dollar (USD)
SEK: Swedish krona
