# Supplementary material for: Experiences of using a digital tool, the D-foot, in the screening of risk factors for diabetic foot ulcers
Source: J Foot Ankle Res. 2022 Dec 13;15:90. doi: 10.1186/s13047-022-00594-9 (PMC9746139; doi:10.1186/s13047-022-00594-9)
Supplement: Supplementary file 1 — Additional file 1. Patient survey included and programmed in the D-Foot. [file 13047_2022_594_MOESM1_ESM.pdf]

Additional file 1

Patient survey included and programmed in the D-Foot

D-Foot patient questions 20160224

No. Heading/question	**Field text in D-Foot**	**Alternative**
2. E-mail		
E-mail	Enter your e-mail	
Confirm	Enter your e-mail again	
3. Telephone number		
Telephone number	Enter your telephone number	
Confirm your telephone number	Enter your telephone number again	
4. Occupation		Work (state your level of employment in %)
		Pensioner
		Student
		Other
5. Height	cm	
6. Weight	kg	
7. In which year were you diagnosed with diabetes?	If you do not know exactly, give an approximate answer	
8. Which type of diabetes do you have?		Diabetes type 1
		Diabetes type 2
9. Which HbA1c value (known as "long-term blood sugar") did you have at your last check-up?	If you do not know exactly, give an approximate answer	
10. Do you smoke?		Yes
		No
11. Do you use snuff?		Yes
		No
12. Do you take medicine for high blood pressure?		Yes
		No

13. Do you take medicine for cardiovascular disease?		Yes<br>No
14. Have you previously received shoes or insoles because of your diabetes?		Yes<br>No
If so, in which year did you make your first visit?	YYYY If you do not know exactly, give an approximate answer	
15. Do you think you can walk normally?		Yes<br>No
16. Do you think you have good balance?		Yes<br>No
17. Do you use walking aids in any context?		Yes<br>No
18. Do you think you have normal feeling in your feet?		Yes<br>No
19. Indicate on the scale how much of the day you sit/lie or stand/walk. This applies to the past week.	A scale from “Sit and lie all day” to<br>“Stand or walk all day”	
During the past 12 months	Relates to Questions 20-22	
20. Have your feet been examined by a doctor or diabetes nurse?		Yes<br>No
21. Have you received podiatry?		Yes<br>No
22. Do you think you have received sufficient information relating to self-care of your feet?		Yes<br>No
Do the healthcare staff think you have reduced circulation in your feet?		Yes<br>No
A. Do you feel numbness/tingling in your feet?		Yes<br>No

B. Do your feet sweat less than before?		Yes<br>No
C. How much pain do you have in your right foot?	A scale from “No pain” to “Extreme pain”	
How much pain do you have in your left foot?	-“-	
D. Have you had ulcers on your right foot?		Yes<br>No
Have you had ulcers on your left foot?		Yes<br>No

Note: In the first column (Nr. Rubrik/Fråga, in English No, Heading/Question), the number and heading of the question is seen, followed by a column (Fälttext in D-Foot, in English field text in D-Foot) describing the text that was programmed in the D-Foot. In the column to the right (Alternativ, in English Alternative), the different alternative answers are presented.
