# Supplementary material for: Experiences of using a digital tool, the D-foot, in the screening of risk factors for diabetic foot ulcers
Source: J Foot Ankle Res. 2022 Dec 13;15:90. doi: 10.1186/s13047-022-00594-9 (PMC9746139; doi:10.1186/s13047-022-00594-9)

Additional file 2, examples of different steps in the D-Foot examination

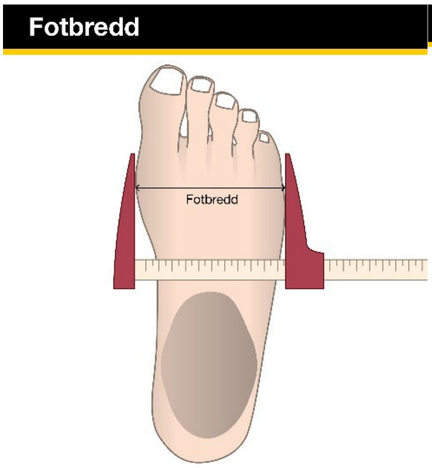

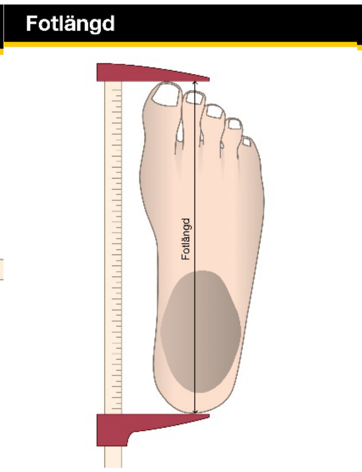

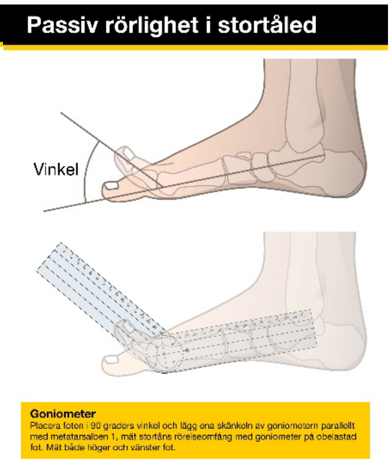

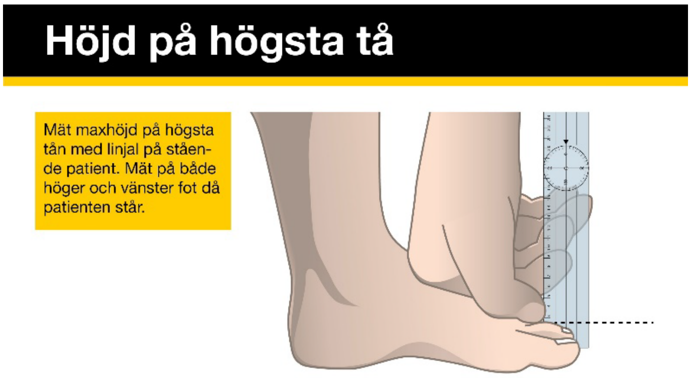

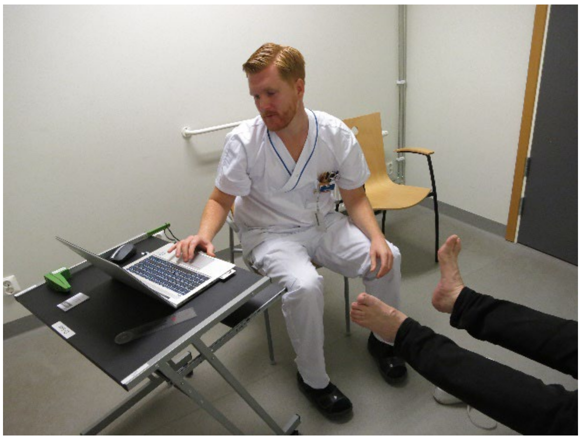

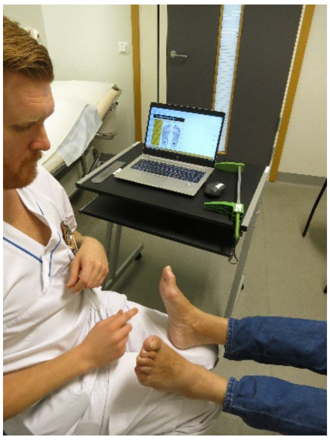

Supplement: Supplementary file 2 — Additional file 2. Examples of different steps in the D-Foot examination. [file 13047_2022_594_MOESM2_ESM.pdf]
