# Supplementary material for: Experiences of using a digital tool, the D-foot, in the screening of risk factors for diabetic foot ulcers
Source: J Foot Ankle Res. 2022 Dec 13;15:90. doi: 10.1186/s13047-022-00594-9 (PMC9746139; doi:10.1186/s13047-022-00594-9)
Supplement: Supplementary file 3 — Additional file 3. Prevention and multidisciplinary service (MDS) of foot complications in diabetes. [file 13047_2022_594_MOESM3_ESM.pdf]

Prevention and multidisciplinary service (MDS) of foot complications in diabetes

Risk grade	Symptoms	Podiatry	Regular foot control made by nurse or doctor	Footwear and orthotic service
1	No signs of distal neuropathy or peripheral vascular disease and no other risk factors	Information of self-care	YES	NO
2	Signs of distal neuropathy or peripheral vascular disease	YES	YES	YES
3	Signs of distal neuropathy or peripheral vascular disease, previous ulceration amputation, foot deformity, skin pathology (e.g. callosities/fissures)	YES	YES	YES
4	Presence of foot ulcer or osteoarthropathy or severe pain syndrome	YES and MDS	YES and MDS	YES and MDS

Illustrations: Pontus Andersson

Risk stratification 1-4 and recommended treatment illustration from the web program D-Foot (www.dfoot.se).
