# Supplementary material for: Experiences of using a digital tool, the D-foot, in the screening of risk factors for diabetic foot ulcers
Source: J Foot Ankle Res. 2022 Dec 13;15:90. doi: 10.1186/s13047-022-00594-9 (PMC9746139; doi:10.1186/s13047-022-00594-9)
Supplement: Supplementary file 4 — Additional file 4. [file 13047_2022_594_MOESM4_ESM.pdf]

1. The Ipswich Touch Test

NOTE!

Ask the patient to close their eyes during the test.

The touch must be light as a feather, and brief (1-2 seconds).

If the patient did not respond, do not try again with harder pressure the second time. Only one try for the 6 toes. If not felt, do not repeat the touch.

Register as YES (sensory loss) if the patient does not have sensory respond ≥2 toes.

If the patient has only one leg, sensory loss is present if the patient does not have sensory ≥1 toe.

(With permission from the designer of The Ipswich Touch Test, Gerry Rayman and the team at Ipswich Hospital).

Touch the toes following the numbering 1 to 6

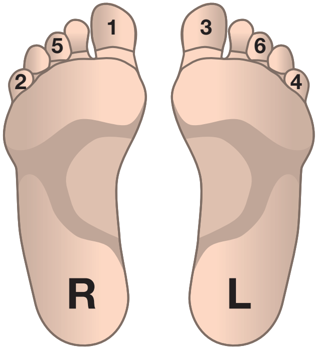

Patient's right foot

Patient's left foot

2. Passive motion at hallux joint

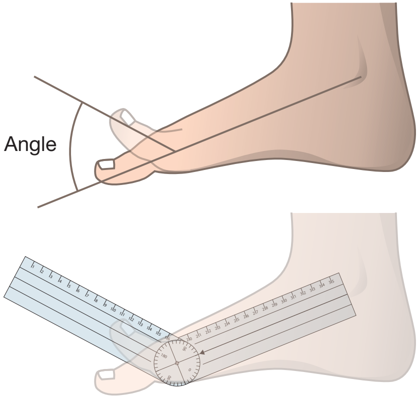

9. Hallux Valgus/Varus

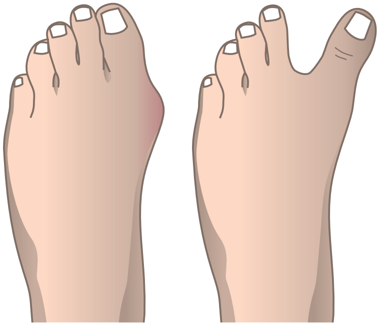

Hallux Valgus

Hallux Varus

12. Calcaneus Valgus/Varus

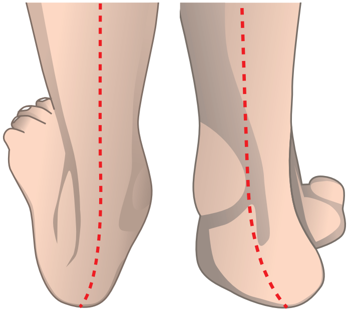

Calcaneus valgus

Calcaneus varus

10. Maximal toe height

Measure maximum height at the highest toe with a ruler on the standing patient. Measure both right and left foot when the patient is standing.

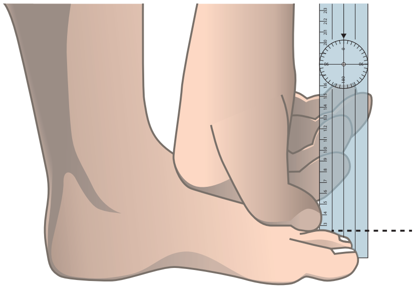

6. Insufficient function of the toes and metatarsal phalangeal joints

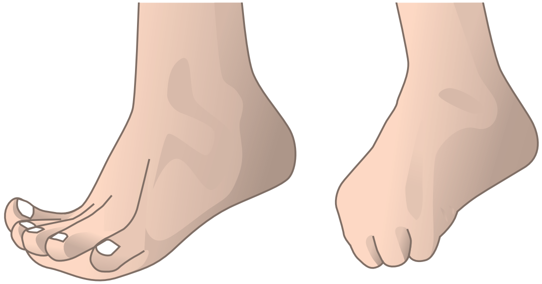

Stretch and flex the toes of both feet.
