# Supplementary material for: Experiences of using a digital tool, the D-foot, in the screening of risk factors for diabetic foot ulcers
Source: J Foot Ankle Res. 2022 Dec 13;15:90. doi: 10.1186/s13047-022-00594-9 (PMC9746139; doi:10.1186/s13047-022-00594-9)
Supplement: Supplementary file 5 — Additional file 5. System Usability Scale answered prior to the study. [file 13047_2022_594_MOESM5_ESM.pdf]

Additional file 5 System Usability Scale answered prior to the study

Appendix 5c_I_start

Questionnaire to orthopaedic engineers/shoe technicians taking part in a study of digital assessments of feet in connection with diabetes using the D-Foot web program

This questionnaire contains questions on what you think it will be like to work digitally, i.e. using a laptop and the D-Foot web program, when you examine the feet of patients with diabetes and then write your documentation. Answer the questions by marking the answer that is most suitable. If you are unsure, you should still mark the alternative that feels most correct. Put a cross in the square like this ☒

What do you think it will be like to work digitally, i.e. using a laptop and the D-Foot web program, when you examine the feet of patients with diabetes and then comment in patient notes?

1) **I think that I would like to make a digital assessment.**

1. Disagree completely	2. Do not agree	3. Neither agree nor disagree	4. Agree	5. Agree completely
☐	☐	☐	☐	☐

2) **I think I will find that a digital foot assessment is unnecessarily complex.**

1. Håller absolut inte med	2. Håller inte med	3. Håller varken med eller inte med	4. Håller med	5. Håller fullkomligt med
☐	☐	☐	☐	☐

3) **I think I will find that a digital foot assessment is easy to perform.**

1. Håller absolut inte med	2. Håller inte med	3. Håller varken med eller inte med	4. Håller med	5. Håller fullkomligt med
☐	☐	☐	☐	☐

4) **I think that I will need the support of a technical person to be able to make a digital assessment.**

1. Håller absolut inte med	2. Håller inte med	3. Håller varken med eller inte med	4. Håller med	5. Håller fullkomligt med
☐	☐	☐	☐	☐

5) **I think I will find that the various functions for performing digital assessments are well integrated.**

1. Håller absolut inte med	2. Håller inte med	3. Håller varken med eller inte med	4. Håller med	5. Håller fullkomligt med
☐	☐	☐	☐	☐

6) **I think I will find that there is too much inconsistency in the digital assessment.**

1. Håller absolut inte med	2. Håller inte med	3. Håller varken med eller inte med	4. Håller med	5. Håller fullkomligt med
☐	☐	☐	☐	☐

7) I think that most people will quickly learn how to make a digital assessment.

1. Håller absolut inte med	2. Håller inte med	3. Håller varken med eller inte med	4. Håller med	5. Håller fullkomligt med
☐	☐	☐	☐	☐

8) **I think I will find making digital assessments very complicated.**

1. Håller absolut inte med	2. Håller inte med	3. Håller varken med eller inte med	4. Håller med	5. Håller fullkomligt med
☐	☐	☐	☐	☐

9) **I think I will feel very confident about making a digital assessment.**

1. Håller absolut inte med	2. Håller inte med	3. Håller varken med eller inte med	4. Håller med	5. Håller fullkomligt med
☐	☐	☐	☐	☐

10) I think I will need to learn a lot of things before I can make a digital assessment.

1. Håller absolut inte med	2. Håller inte med	3. Håller varken med eller inte med	4. Håller med	5. Håller fullkomligt med
☐	☐	☐	☐	☐
