# Supplementary material for: Experiences of using a digital tool, the D-foot, in the screening of risk factors for diabetic foot ulcers
Source: J Foot Ankle Res. 2022 Dec 13;15:90. doi: 10.1186/s13047-022-00594-9 (PMC9746139; doi:10.1186/s13047-022-00594-9)
Supplement: Supplementary file 6 — Additional file 6. System Usability Scale answered after the study. [file 13047_2022_594_MOESM6_ESM.pdf]

Additional file 6 System Usability Scale answered after the study

Questionnaire to orthopaedic engineers´/shoe technicians taking part in a study of foot examinations at a Department of Prosthetics and Orthotics and the implementation of the D-Foot

This questionnaire contains questions on foot examinations you have performed on patients with diabetes and foot complications. Answer the questions by marking the answer that is most suitable. If you are unsure, you should still mark the alternative that feels most correct. Put a cross in the square like this ☒

Part I: What was it like to use a tablet and answer the questions in the D-Foot web program?

1) **I think I shall use the D-Foot frequently.**

1. Håller absolut inte med	2. Håller inte med	3. Håller varken med eller inte med	4. Håller med	5. Håller fullkomligt med
☐	☐	☐	☐	☐

2) **I thought that the D-Foot was unnecessarily complicated.**

1. Håller absolut inte med	2. Håller inte med	3. Håller varken med eller inte med	4. Håller med	5. Håller fullkomligt med
☐	☐	☐	☐	☐

3) **I thought the D-Foot was easy to use.**

1. Håller absolut inte med	2. Håller inte med	3. Håller varken med eller inte med	4. Håller med	5. Håller fullkomligt med
☐	☐	☐	☐	☐

4) **I needed technical assistance to use the D-Foot.**

1. Håller absolut inte med	2. Håller inte med	3. Håller varken med eller inte med	4. Håller med	5. Håller fullkomligt med

☐	☐	☐	☐	☐

5) **I thought the different functions in the D-Foot worked well.**

1. Håller absolut inte med	2. Håller inte med	3. Håller varken med eller inte med	4. Håller med	5. Håller fullkomligt med
☐	☐	☐	☐	☐

6) **I thought the design of the D-Foot was illogical.**

1. Håller absolut inte med	2. Håller inte med	3. Håller varken med eller inte med	4. Håller med	5. Håller fullkomligt med
☐	☐	☐	☐	☐

7) **I think most people will quickly learn to use the D-Foot.**

1. Håller absolut inte med	2. Håller inte med	3. Håller varken med eller inte med	4. Håller med	5. Håller fullkomligt med
☐	☐	☐	☐	☐

8) **I thought that using the D-Foot was complicated.**

1. Håller absolut inte med	2. Håller inte med	3. Håller varken med eller inte med	4. Håller med	5. Håller fullkomligt med
☐	☐	☐	☐	☐

9) **Using the D-Foot felt secure.**

1. Håller absolut inte med	2. Håller inte med	3. Håller varken med eller inte med	4. Håller med	5. Håller fullkomligt med
☐	☐	☐	☐	☐

10) **I needed to learn many new things before I was able to start using the D-Foot.**

1. Håller absolut inte med	2. Håller inte med	3. Håller varken med eller inte med	4. Håller med	5. Håller fullkomligt med
☐	☐	☐	☐	☐
