# Supplementary material for: Experiences of using a digital tool, the D-foot, in the screening of risk factors for diabetic foot ulcers
Source: J Foot Ankle Res. 2022 Dec 13;15:90. doi: 10.1186/s13047-022-00594-9 (PMC9746139; doi:10.1186/s13047-022-00594-9)
Supplement: Supplementary file 7 — Additional file 7. Questions related to the information the certified prosthetist and orthotist gave to the patients and questions related to integrity and time to finalise the visit. [file 13047_2022_594_MOESM7_ESM.pdf]

Additional file 7 Questions related to the information the certified prosthetist and orthotist gave to the patients and questions related to integrity and time to finalise the visit

Part II: Describe your reception

Answer the questions by marking the answer that is most suitable on a scale of 1 (very poor or not at all) to 4 (very good or Yes, completely). If you are unsure, you should still mark the alternative that feels most correct. Put a cross in the square like this ☒

11) **Did the patient receive information about how to perform self-care of the feet?**

1. Not at all	2.	3.	4. Yes, completely	5. Not relevant
☐	☐	☐	☐	☐

12) **Was the patient's illness/health condition discussed with the patient?**

1. Inte alls	2.	3.	4. Ja, helt och hållet	5. Ej aktuellt
☐	☐	☐	☐	☐

13) **Did you provide information about where to go if the patient needed help or had additional questions after the visit?**

1. Inte alls	2.	3.	4. Ja, helt och hållet	5. Ej aktuellt
☐	☐	☐	☐	☐

14) **Did the patient receive information about possible risks when using the assistive device?**

1. Inte alls	2.	3.	4. Ja, helt och hållet	5. Ej aktuellt
☐	☐	☐	☐	☐

15) **Did the patient receive information about warning signs to be aware of regarding his/her illness/health condition or the assistive device?**

1. Inte alls	2.	3.	4. Ja, helt och hållet	5. Ej aktuellt
☐	☐	☐	☐	☐

16) **Was there enough privacy when you and the patient discussed his/her condition or treatment?**

1. Inte alls	2.	3.	4. Ja, helt och hållet	5. Ej aktuellt
☐	☐	☐	☐	☐

17) **Did you explain the results of the D-Foot survey to the patient?**

1. Inte alls	2.	3.	4. Ja, helt och hållet	5. Ej aktuellt
☐	☐	☐	☐	☐

18) **Did the patient receive oral user information?**

1. Inte alls	2.	3.	4. Ja, helt och hållet	5. Ej aktuellt
☐	☐	☐	☐	☐

19) **Did the patient receive written user information?**

1. Inte alls	2.	3.	4. Ja, helt och hållet	5. Ej aktuellt
☐	☐	☐	☐	☐

20) **How long did the patient visit last? Do not include the time taken to enter information on the visit in Pilot.**

☐ Less than 30 minutes

☐ 31-45 minutes

☐ 46-60 minutes

☐ More than 60 minutes

21) **How long did it take to enter information in Pilot when you examined feet according to the D-Foot method?**

☐ Less than 5 minutes

☐ 6-10 minutes

☐ 11-15 minutes

☐ 16-20 minutes

22) **How long did it take to make any shoe and material orders?**

☐ Less than 5 minutes

☐ 6-10 minutes

☐ 11-15 minutes

☐ 16-20 minutes
