# Supplementary material for: Experiences of using a digital tool, the D-foot, in the screening of risk factors for diabetic foot ulcers
Source: J Foot Ankle Res. 2022 Dec 13;15:90. doi: 10.1186/s13047-022-00594-9 (PMC9746139; doi:10.1186/s13047-022-00594-9)
Supplement: Supplementary file 8 — Additional file 8. [file 13047_2022_594_MOESM8_ESM.pdf]

Additional file 8

20) **How long did the patient visit last? Do not include the time taken to enter information on the visit in Pilot.**

☐ Less than 30 minutes

☐ 31-45 minutes

☐ 46-60 minutes

☐ More than 60 minutes

21) **How long did it take to enter information in Pilot when you have examined the feet according to the D-Foot method?**

☐ Less than 5 minutes

☐ 6-10 minutes

☐ 11-15 minutes

☐ 16-20 minutes

22) **How long did it take to order any shoes and materials?**

☐ Less than 5 minutes

☐ 6-10 minutes

☐ 11-15 minutes

☐ 16-20 minutes
