# Supplementary figures and images for: Experiences of using a digital tool, the D-foot, in the screening of risk factors for diabetic foot ulcers
Source: J Foot Ankle Res. 2022 Dec 13;15:90. doi: 10.1186/s13047-022-00594-9 (PMC9746139; doi:10.1186/s13047-022-00594-9)

35 shore

55 shore

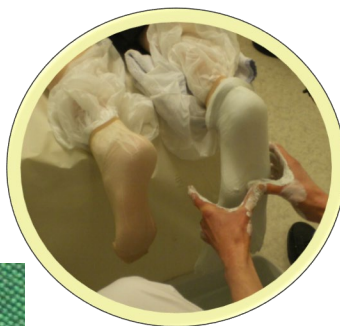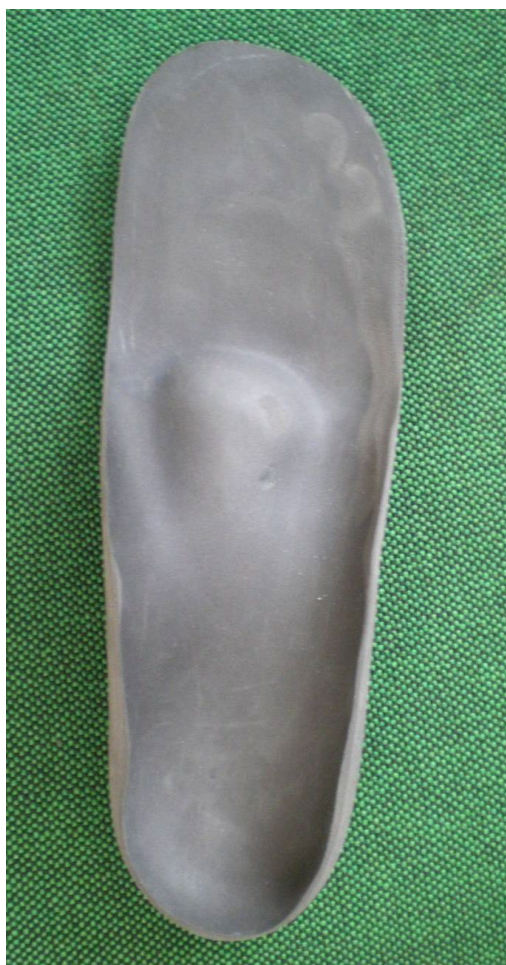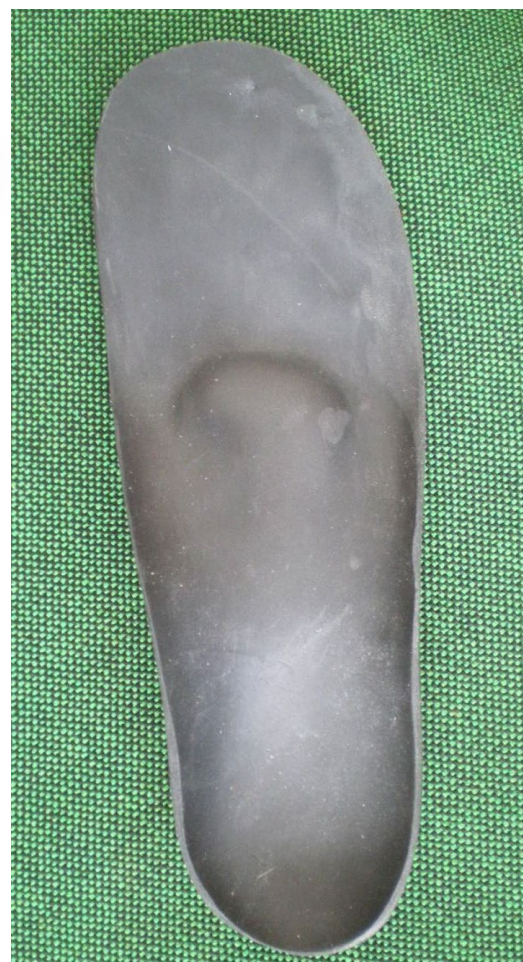

Supplement: Supplementary file 9 — Additional file 9. [file 13047_2022_594_MOESM9_ESM.pdf]
