# Supplementary material for: Experiences of using a digital tool, the D-foot, in the screening of risk factors for diabetic foot ulcers
Source: J Foot Ankle Res. 2022 Dec 13;15:90. doi: 10.1186/s13047-022-00594-9 (PMC9746139; doi:10.1186/s13047-022-00594-9)
Supplement: Supplementary file 10 — Additional file 10. [file 13047_2022_594_MOESM10_ESM.pdf]

Patient 19121212-1212

Riskgrad: 3

Prevention och multidisciplinär service (MDS) vid fotkomplikation, diabetes

Riskgrad	Symtom	Fotvård	Årlig fotundersökning hos läkare eller diabetessjuksköterska	Ortopedteknisk behandling
3	Tecken på distal neuropati eller kärlsjukdom, förekommit fotsår/ amputation, fotdeformitet, hudpatologi (t.ex. callus/fissur)	JA	JA	JA

Patientenkät	Svar
Min sysselsättning	Jobbar
Min längd	175
Min vikt	65
Min bmi	21
Vilket år fick du diagnosen diabetes?	2022
Vilken typ av diabetes har du?	Diabetes typ 1
Vilket HbA1c-värde (kallat långsocker) hade du vid din senaste kontroll?	62
Röker du?	Ja
Snusar du?	Ja
Tar du medicin för högt blodtryck?	Ja
Tar du medicin för hjärt-kärlssjukdomar	Ja
Har du tidigare fått skor eller inlägg på grund av din diabetssjukdom?	Ja
I så fall, vilket år gjorde du ditt första besök?	2022
I så fall, var (vilken mottagning) fick du skor eller inlägg?	här
Anser du att du kan gå normalt?	Nej
Upplever du att du har normal känsel i fötterna?	Nej
Markera på skalan hur stor del av dagen som du sitter/ligger eller står/går. Markeringen på skalan avser den senaste veckan.	
Har dina fötter blivit undersökta av läkare eller diabetssjuksköterska under de senaste 12 månaderna?	Nej
Har du fått fotvård under de senaste 12 månaderna?	Nej
Anser du att du fått tillräcklig information om egenvård av fötterna under de senaste 12 månaderna?	Nej
**Har vårdpresonal konstaterat att du har nedsatt blodcirkulation i fötterna?**	**Nej**
**Känner du domningar/pirrningar i fötterna?**	**Ja**
**Har du mindre fotsvett nu än tidigare?**	**Ja**
Hur mycket smärta har du i höger fot?	
Hur mycket smärta har du i vänster fot?	
**Har du haft sår på höger fot?**	**Ja**
**Har du haft sår på vänster fot?**	**Ja**

Undersökning	Vänster	Höger
**Amputation ovan fotled**	**Nej**	**Nej**
**Amputation under fotled**	**Nej**	**Nej**
**Sår**	**Nej**	**Nej**
**Akut Charcot fot**	**Nej**	**Nej**

**Charcot fot**	**Nej**	**Nej**
**Känselbortfall, Ipswich Touch Test**	**Ja**	**Ja**
**Plantart ytliga ben och ledstrukturer**	**Ja**	**Ja**
Har patienten förhårdnader?		
**Förhårdnader**	**Medialt**	**Medialt**
**Inspektion av fotbeklädnad. Har bakkappan förskjutits?**	**Medialt**	**Medialt**
**Inspektion av fotbeklädnad. Är slitsulan nedsliten?**	**Medialt**	**Medialt**
**Maximal passiv dorsalextension i stortåleden**	**25**	**40**
**Hallux valgus/varus**	**Valgus**	**Valgus**
**Medial överbelastning**	**Ja**	**Ja**
**Lateral överbelastning**	**Ja**	**Ja**
Adduktion av framfoten	Nej	Nej
Abduktion av framfoten	1	1
**Maximal tåhöjd på högsta tån**	**25**	**32**
Fotledsrörlighet	25	30
Fotlängd	266	260
Fotbredd	110	105
**Fotisättning vid gående**	**Häl först**	**Häl först**

(Fetstil indikerar faktor som påverkar riskgrad)
